# Machine learning prediction of stone-free success in patients with urinary stone after treatment of shock wave lithotripsy

**DOI:** 10.1186/s12894-020-00662-x

**Published:** 2020-07-03

**Authors:** Seung Woo Yang, Yun Kyong Hyon, Hyun Seok Na, Long Jin, Jae Geun Lee, Jong Mok Park, Ji Yong Lee, Ju Hyun Shin, Jae Sung Lim, Yong Gil Na, Kiwan Jeon, Taeyoung Ha, Jinbum Kim, Ki Hak Song

**Affiliations:** 1 Department of Urology, Chungnam National University College of Medicine, Chungnam National University Hospital, 282 Monwha-ro, Jung-gu Daejeon, Republic of Korea 35015; 2 Division of Medical Mathematics, National Institute for Mathematical Sciences, 70 Yuseong-daero 1689beon-gil, Yuseong-gu Daejeon, Republic of Korea 34047; 3 Department of Urology, Konyang University College of Medicine, Konyang University Hospital, 158 Gwanjeodong-ro, Seo-gu Daejeon, Republic of Korea 35365

**Keywords:** Lithotripsy, Machine learning, Artificial intelligence

## Abstract

**Background:**

The aims of this study were to determine the predictive value of decision support analysis for the shock wave lithotripsy (SWL) success rate and to analyze the data obtained from patients who underwent SWL to assess the factors influencing the outcome by using machine learning methods.

**Methods:**

We retrospectively reviewed the medical records of 358 patients who underwent SWL for urinary stone (kidney and upper-ureter stone) between 2015 and 2018 and evaluated the possible prognostic features, including patient population characteristics, urinary stone characteristics on a non-contrast, computed tomographic image. We performed 80% training set and 20% test set for the predictions of success and mainly used decision tree-based machine learning algorithms, such as random forest (RF), extreme gradient boosting trees (XGBoost), and light gradient boosting method (LightGBM).

**Results:**

In machine learning analysis, the prediction accuracies for stone-free were 86.0, 87.5, and 87.9%, and those for one-session success were 78.0, 77.4, and 77.0% using RF, XGBoost, and LightGBM, respectively. In predictions for stone-free, LightGBM yielded the best accuracy and RF yielded the best one in those for one-session success among those methods. The sensitivity and specificity values for machine learning analytics are (0.74 to 0.78 and 0.92 to 0.93) for stone-free and (0.79 to 0.81 and 0.74 to 0.75) for one-session success, respectively. The area under curve (AUC) values for machine learning analytics are (0.84 to 0.85) for stone-free and (0.77 to 0.78) for one-session success and their 95% confidence intervals (CIs) are (0.730 to 0.933) and (0.673 to 0.866) in average of methods, respectively.

**Conclusions:**

We applied a selected machine learning analysis to predict the result after treatment of SWL for urinary stone. About 88% accurate machine learning based predictive model was evaluated. The importance of machine learning algorithm can give matched insights to domain knowledge on effective and influential factors for SWL success outcomes.

## Background

Shock wave lithotripsy (SWL), which was first introduced by Chaussy in 1980 [1], has been recognized as convenient, noninvasive management for urinary stones, and now it is widely used as primary treatment for urinary stones smaller than 2 cm sized due to high stone-free rate [2–4]. However, the stone-free rate of SWL in the treatment of urinary stone is affected by the size, location, composition, and radiological density of the stone. Especially in cases of large stones, the success rate is relatively low, and the rate of retreatment is high, thus requiring more time and resulting in low cost-effectiveness [5].

Ineffective procedures can be avoided, and unnecessary resource waste can be prevented by choosing better treatment methods for stone management by evaluating whether patients with urinary stones can respond well to SWL or not. The popular use of non-contrast computed tomography (NCCT) in the diagnosis of urinary stone has allowed accurate measurement of stone characteristics such as size, shape, location, and consistency, using Hounsfield units (HU). Therefore, considering the factors that may affect the stone-free rate, it is possible to reduce such retreatment and economic costs by selectively applying SWL. Many researchers have tried to determine these factors by using statistical methods, and various studies have been reported to predict the stone-free rate after SWL [6–8].

Recently, the importance of machine learning and artificial intelligence technology has increased, and get more attention in medical areas with the advent of big data. In the medical field, researchers applied machine learning methodology to various disease diagnoses and predictions [9, 10]. The purposes of this study are to investigate retrospective information on patients with a diagnosis of urinary stone who underwent SWL and to establish a machine learning model in binary classification for predicting the stone-free, or not and one-session success, or not after SWL. Furthermore, the resulting machine learning prediction models can be implemented as an actual diagnostic support system for urinary stone treatment and provide more capability or new functionality for it.

## Methods

We retrospectively identified patients with kidney and upper-ureter stones who underwent the first start session of SWL at our institution between January 2015 and December 2018. All data analysis was carried out in accordance with applicable laws and regulations described in the Declaration of Helsinki and approved by Chungnam national university hospital institutional review board approval (CNUH 2018–07-047). Three hundred fifty-eight patients with previously untreated stones and a solitary stone diameter of 5 to 20 mm were included. Patients were excluded if they were younger than 18 years old; had a congenital genitourinary tract anomaly, history of previous open urinary-tract surgery, or multiple stones, or who had not undergone imaging for 4 weeks after SWL. We retrospectively reviewed the medical records and picture archiving and communication system (PACS) data of these patients and evaluated the possible prognostic features, including age and sex; presence of diabetes mellitus (DM) or hypertension (HTN); stone characteristics such as stone laterality, location, maximal length, stone volume, skin to stone distance (SSD), mean stone density (MSD), and stone heterogeneity index (SHI); double-J stenting and percutaneous nephrostomy (PCN) procedure before SWL; simple psoas muscle cross-sectional area measurement for sarcopenia; complete blood cell count; liver function test; renal function test; electrolyte test; and urinalysis.

### Stone characteristics on NCCT

The stone characteristics were interpreted by NCCT, and each maximal length was measured on axial, coronal, and sagittal NCCT scan. The volume of the stone was computed by the ellipsoid method (X-axis length x Y-axis length x Z-axis length x π/6). The SSD was calculated using radiographic calipers from the point of the largest stone diameter at 90^o^ from the horizontal plane because of vertical shockwave delivery through the patient’s back. HU was carefully calculated on the magnified, axial NCCT image from a circle with a diameter of about 2–3 mm in the center of the middle cross-section without including the adjacent tissue. MSD was identified as the mean value of HU, and SHI was identified as the standard deviation of HU.

### SWL protocol

SWL was performed on an outpatient basis, without anesthesia. The same lithotripter was used to treat all patients, with fluoroscopic guidance. The lithotripter was an electromagnetic lithotripter made by the DirexGroup (Integra SL, Initia Ltd., Israel). The intensity of the shock wave started from 10.0 kV and gradually increased less than 18.0 kV to improve stone fragmentation and reduce the risk of adjacent tissue trauma. The number of shock waves per SWL session varied from 2300 to 2500 at a rate of 60 shock waves per minute.

Additional SWL was performed at intervals of 1 week if evidence of stones remained. The stone-free was defined as the absence of observed stone with X-ray studies or asymptomatic condition and clinically insignificant residual fragments ≤3 mm in maximal length 4 weeks after the first SWL treatment as measured by simple abdominal radiography or NCCT. One-session success was defined as patients who were stone-free after a single SWL treatment. Enough water intake and appropriate exercise were recommended for all patients.

### Data analytics

The SWL data has 42 features including the two target variables, stone-free and one- session success, and a total of 358 cases. The SWL data were analyzed using well-known machine learning methods such as random forest (RF), which is a statistical machine learning method [11] and extreme gradient boosting trees (XGBoost), which is a decision tree–based gradient boosting regression method [12] and light gradient boosting method (LightGBM) [13]. The machine learning models were trained in binary classifications for predicting the targets, stone-free and one-session success. Experiments were performed with 80% of the data for training the prediction model (training set) and 20% for testing the trained model (test set). For the experiment, we randomly sampled 10 times, and then take average of its results, which is similar to n-fold validation method, and performed the prediction for stone-free and one-session success. The sampling strategy shows a certain capability of predictive models obtained from the given SWL data set. For calculating sensitivity, specificity, PPV, we computed the confusion matrix and do computed their values in average over sample data sets for prediction tests, and AUCs, we adopted sklearn.metric module in Python. For 95% CIs, we used bootstrapping methods with 1000 bootstraps for each sampled data sets, and then took the average over samples.

## Results

The number of cases with stone-free and one-session success were 253 (70.7%) and 154 (43.0%). Table 1 shows the patient and stone characteristics of the patient data, which we used for predictions. We present the prediction accuracies in Table 2. The prediction accuracies for the stone-free were 86.0, 87.5%, and 87.9 and those for one-session success were 78.0, 77.4, and 77.0% using RF, XGBoost, and LightGBM, respectively. In predictions for the stone-free, LightGBM offers better accuracy than XGBoost and RF; and for one-session success, RF algorithms showed better accuracy than XGBoost and LightGBM.

**Table 1:** General characteristics of all urinary stone patients

	Kidney stone	Ureter stone	*P*-value
Patient characteristics			
Numbers of patients	167	191	
Age, mean ± SD	56.4 ± 13.9	60.4 ± 13.7	0.007
Sex, numbers of men, %	90, 53.9	108, 56.5	0.615
Diabetes mellitus, %	48, 28.7	57, 29.8	0.820
Hypertension, %	65, 38.9	86, 45.0	0.243
Psoas muscle cross-sectional area (mm^2^), mean ± SD	1105.9 ± 373.1	1067.4 ± 368.7	0.326
Stone characteristics			
Stone laterality, numbers on left side, %	87, 52.1	93, 48.7	0.520
Stone length (mm, X-axis), mean ± SD	9.2 ± 3.5	6.6 ± 1.5	< 0.001
Stone length (mm, Y-axis), mean ± SD	9.3 ± 3.2	7.4 ± 1.7	< 0.001
Stone length (mm, Z-axis), mean ± SD	9.7 ± 3.1	9.4 ± 2.8	0.345
Stone volume (mm^3^), mean ± SD	516.8 ± 479.6	268.2 ± 182.2	< 0.001
Skin to stone distance 90^o^ (mm), mean ± SD	87.9 ± 14.8	108.9 ± 16.3	< 0.001
Mean stone density, mean ± SD	834.3 ± 296.9	766.6 ± 274.7	0.025
Stone heterogeneity index, mean ± SD	178.0 ± 83.9	174.2 ± 84.9	0.669

*SD* standard deviation

**Table 2:** Comparison of prediction accuracies for stone-free and one-session success according to three machine learning methods

	Stone-free	One-session success
Random forest (RF)		
Training Accuracy (%)	86.47	76.83
Test Accuracy (%)	85.98	78.02
Extreme gradient boosting trees (XGBoost)		
Training Accuracy (%)	87.50	75.60
Test Accuracy (%)	87.46	77.39
Light Gradient Boosting Method (LightGBM)		
Training Accuracy (%)	88.09	74.92
Test Accuracy (%)	87.95	77.04

**Fig. 1:**
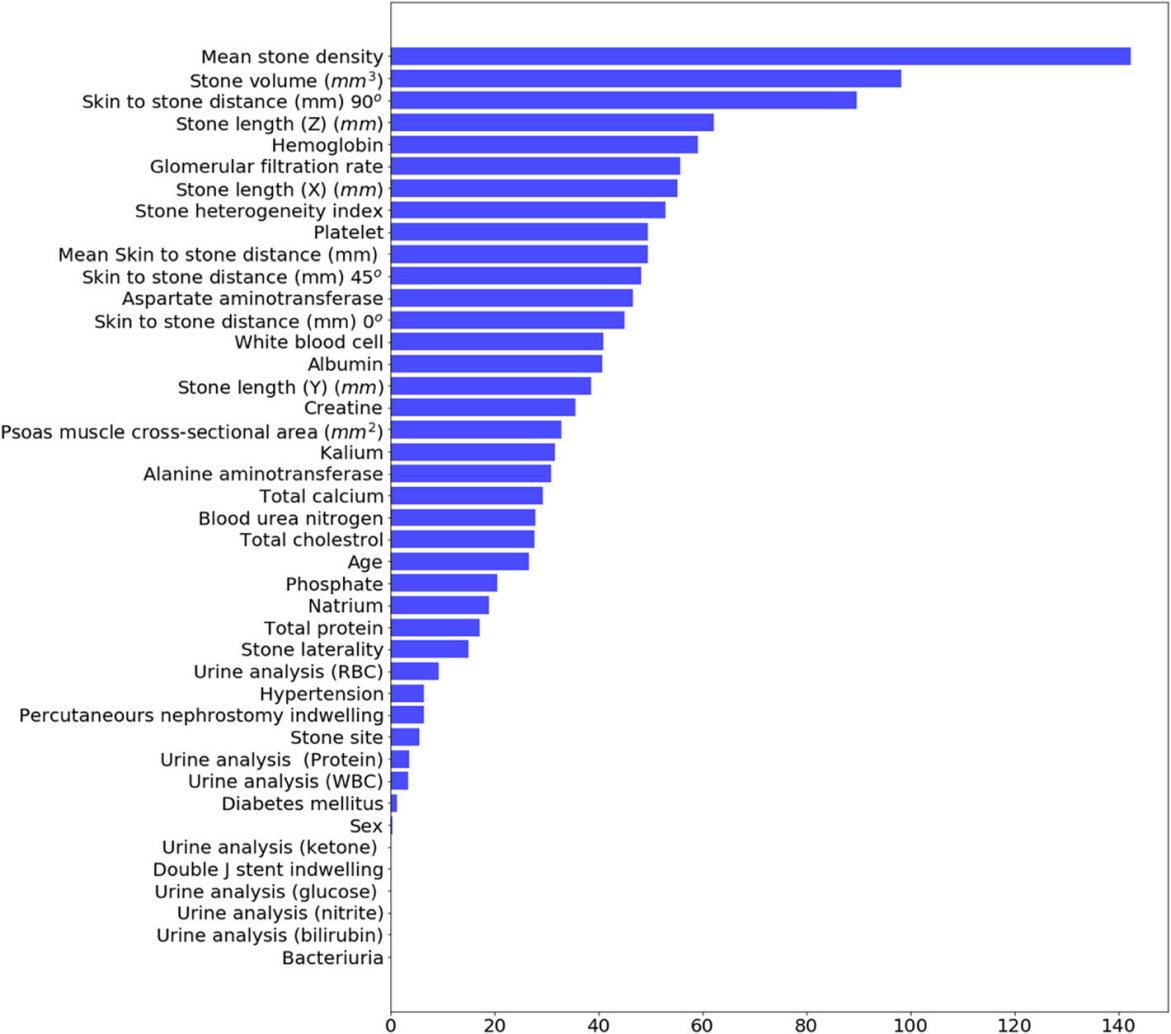
Feature importance of LightGBM for stone-free prediction. Stone-free was affected with mean stone density, stone volume, and skin to stone distance

**Fig. 2:**
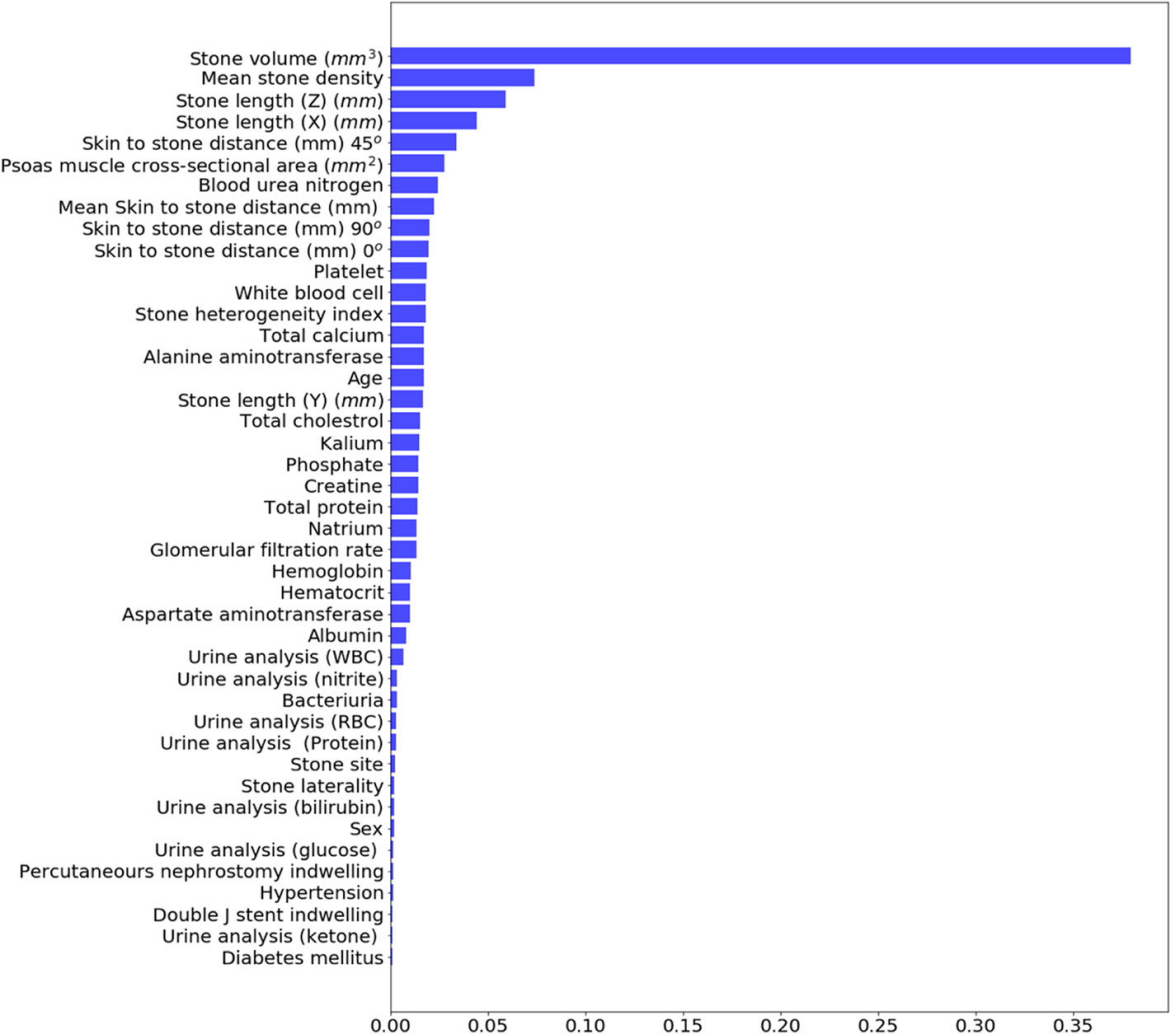
Feature importance of Random Forest for one-session success prediction. One-session success was affected with stone volume, mean stone density, stone length, skin to stone distance, and psoas muscle cross-sectional area

The sensitivity, specificity, positive predictive value, confidence interval and area under the roc curve (AUC) for machine learning analytics are presented in Table 3. As the result in Table 3, the specificity of stone-free was better than the sensitivity. The result implied that the predictive models were more accurate in prediction stone-free. On the contrary, the models were good in the prediction of one-session fail. In AUC for the stone-free, RF and LightGBM offered higher value than XGBoost; and for one-session success, RF offered higher value than XGBoost and LightGBM. Here, we also have shown the feature importance, which had certain interpretability of prediction results related to domain insights. In Fig. 1, we present the feature importance of LightGBM for stone-free predictions. It shows that MSD was the main factor that had decided the stone-free and stone volume (mm^3^) and SSD 90^o^ (mm) played an important role in supporting the stone-free decision. It might match a typical intuition of stone-free; however, with the machine learning method, LightGBM caught the intuition for that. In addition, in the prediction of one-session success, stone volume (mm^3^) was the main factor (Fig. 2).

**Table 3:** Comparison of Receiver Operating Characteristic (ROC) values for stone-free and one-session success according to three machine learning methods

	Stone-free	One-session success
Random forest (RF)		
Sensitivity	0.74	0.81
Specificity	0.92	0.75
AUC	0.85	0.78
CI (95%)	(0.75–0.94)	(0.67–0.86)
PPV	0.82	0.79
Extreme gradient boosting trees (XGBoost)		
Sensitivity	0.75	0.80
Specificity	0.93	0.75
AUC	0.84	0.77
CI (95%)	(0.74–0.93)	(0.68–0.87)
PPV	0.78	0.79
Light Gradient Boosting Method (LightGBM)		
Sensitivity	0.78	0.79
Specificity	0.92	0.74
AUC	0.85	0.77
CI (95%)	(0.73–0.93)	(0.67–0.87)
PPV	0.81	0.78

*AUC* area under ROC curve, *CI* confidence interval, *PPV* positive predictive value

## Discussion

To be able to predict the result of treatment and the patient’s condition with easy measurement would be beneficial for all concerned. The prediction could provide the ability to choose an effective treatment for urinary tract stones and reduce unnecessary resource waste. One promising approach to obtain an appropriate prediction is to adopt machine learning based artificial intelligence methods.

The machine learning methods can show the potential to make decisions that are best suited to the situation without the involvement of emotions, based solely on thorough statistics and calculations. There are some advantages to analyzing data by using machine learning methods. First, machine learning can provide interpretability for analysis and prediction results. Second, insufficient number of data set to apply deep learning algorithms can be handled by certain types of machine learning algorithms, such as tree-based ones; statistical machine learning especially is more effective in predicting with small data, even though more data usually yield more accuracy. Third, machine learning treats heterogeneous data, which is statistically and structurally quite different, simultaneously; for instance, data about kidney stone and ureter stone properties differ distinctly [14, 15]. Machine learning could find new values from data and derives important factors for predicted target variables from the analytical/predicting perspective of machine learning. These factors can be used to validate known domain insights and sometimes reveal factors that were not previously recognized. The main factors of them derived from machine learning are expected to play a major role in developing an auxiliary system for diagnosing diseases and a supporting system.

In this study, data on SWL is accumulated in the treatment of urinary stone, and valid data is available, and we applied three well-known tree-based machine learning methods and compared their results. The decision tree-based methods, such as RF, XGBoost, and LightGBM increased the interpretability of the predicted results by providing the importance of the properties used in the prediction, suggesting new functionality of the machine learning methodology. The reason, why we applied the above decision tree-based machine learning algorithms rather than well-known deep learning ones, is that the tree-based predictive models give better performance in prediction accuracy in case of the relatively small number of data for deep learning ones, in general. Moreover, the algorithms give certain interpretation of their results in feature importance. Another strategy to overcome the relatively small number of data, we collected SWL treatment data for both the upper ureter and kidney stones without their positional information. Then the trained machine learning model with the collected data can show the capability of prediction for stone-free and one-session success. Even though the positional information for urinary stones is neglected, the predictive model can catch the effective accuracy in predictions. After filtering the missing data, carefully, three hundred and fifty-eight cases were obtained for applying machine learning algorithms. The experiment results obtained by taking an average of ten samplings of the given data set. In these prediction experiments, the parameter tuning was not performed for comparison purpose, because parameter tuning shows different results for different sampling of given data.

The major contribution of this study was to enable urologists to choose patients who would realize the most optimal results from SWL. After prediction analysis, patients who have a high risk of stone-free failure can select another method, such as percutaneous nephrolithotomy or retrograde intrarenal surgery, using flexible ureteroscopy to manage urinary stone. The first objective of this study was achieved because each outcome in the predictive analysis exceeded 85% for stone-free and 77% for one-session success, especially, LightGBM and XGBoost showed good prediction outcomes of more than 87% in stone-free prediction.

In most cases of the SWL, the stone analysis could not be done without analyzing the stone fragments that had been discharged from the body directly. Therefore, patients and stone characteristics should have an important role in the pretreatment prediction of treatment outcomes. Even though stone volume, MSD, and SSD are known as important factors that can affect the stone-free rate after SWL, controversy about SSD still exists [16, 17]. In our feature importance analysis, as with the results from various other studies that have been analyzed using general statistical methods, MSD and stone volume were the most influential factors, and SSD was less affected than MSD and stone volume.

MSD is the mean value of the HU of each pixel in a specific stone area and is known as a potential predictor of successful treatment of urinary stone with SWL [18–20]. Eisner et al. found that by measuring the mean HU of defined regions just smaller than the stone in magnified images on each slice of the transverse planes with a standard bone window was the most accurate method of determining MSD [21]. In addition, PACS may provide pixel statistics such as minimum, maximum, and standard deviation of HU values. Lee et al. [22] defined SHI as the standard deviation of stone density on NCCT and assessment that SHI was independently associated with SWL success in patients with ureter stone. In our study, we easily determined MSD by measuring the mean HU from NCCT by using a PACS in the same way. It was significant that MSD was a more important feature than SHI in the prediction of stone-free. All things are taken together, although the predictive level of SHI seems to be lower than MSD, SHI can play a supplementary role in the prediction of SWL treatment outcomes.

The question of whether body mass index (BMI) affects the success rate of SWL treatment has been a controversial issue. Most of the studies have shown that BMI was an independent predictor of stone-free status after SWL [23, 24]. However, several studies took a different view [25, 26], so we tried to think about muscle mass, which is a factor that can indicate the whole-body health condition. No previous study has considered the relationship between muscle mass and the success rate of SWL treatment. Sarcopenia brings about mobility limitation and an inability to perform simple activities of daily life [27]. The psoas muscle cross-sectional area has been used in many studies to provide estimates of overall muscle mass and has been shown to be a simple and easily performed measure of a reliable marker of sarcopenia [28–30]. In this study, we found that the psoas muscle cross-sectional area was ranked as an important feature in the prediction of one-session success rather than in those of stone-free. That is, the higher the muscle mass, the higher the activity in daily life. Muscle mass can be regarded as an important factor in determining the extent of stone removal for a short period.

The results of blood and urine tests showed a generally low feature ranking. Among them, hemoglobin, glomerular filtration rate, and platelet count were judged to give a little meaning.

In the current study, there were some limitations. Its retrospective design may have introduced sampling bias. To compensate for its retrospective sampling bias and small sample size, we applied three machine learning methods, which can reduce bias from commonly used general statistical accesses. However, further studies with prospective data are needed to prove our monitoring on the relationship of feature importance.

MSD was the most significant in feature importance, and stone volume, SSD and stone length were the next most closely associated with stone-free prediction of SWL treatment outcomes in patients with urinary stone. In addition, stone volume was the most significant in feature importance, and MSD, stone length, SSD and psoas muscle cross-sectional area were the next most closely associated with the one-session success prediction in this study. Thus, these would be clinically useful parameters in order.

## Conclusions

We analyzed the effect of SWL treatment by using three machine learning methods and confirmed that prediction accuracy can rise up to as much as 87.9% by using various patients and stone characteristics. We propose that the new machine learning based artificial intelligence and medical encounter are important. When further large studies, validated in a prospective group of urinary stone patients, become available, our machine learning methods might be useful for guiding SWL treatment selection and prediction of patients with urinary stone.

## Data Availability

The datasets used and/or analysed during the current study are available from the corresponding author on reasonable request.

## Abbreviations

SWL: Shock wave lithotripsy
NCCT: Non-contrast computed tomography
HU: Hounsfield units
PACS: Picture archiving and communication system
DM: Diabetes mellitus
HTN: Hypertension
SSD: Skin to stone distance
MSD: Mean stone density
SHI: Stone heterogeneity index
PCN: Percutaneous nephrostomy
RF: Random forest
XGBoost: Extreme gradient boosting trees
LightGBM: Light gradient boosting method
AUC: Area under the roc curve
PPV: Positive predictive value
BMI: Body mass index
